# Foreign Correspondence

**Published:** 1890-02

**Authors:** W. D. Miller


### Foreign Correspondence.

---

To the Editor:

*Concerning the Dental Section of the Tenth International Medical Congress.*—In response to a call of the Organizing Committee (Professors Virchow, Von Bergmann, and Waldeyer), fifty delegates from the various universities and medical societies of Germany met in Heidelberg on the 17th of September, 1888, to take steps in the organization of the congress. At the meeting it was decided that the congress should be held in Berlin, beginning August 4 and closing August 10, 1890.

An organizing committee, consisting of Professors Virchow, Von Bergmann, Leyden, and Waldeyer, was elected, and a general secretary, Dr. Lassar, appointed.

Eighteen sections, including Dental Surgery, were organized, each with a special committee of nine members.

An international medico-scientific exhibition is to be connected with the congress. Statutes and a programme were adopted, which will be given in as far as they particularly concern the Dental Section.

"Art. II. The congress consists of physicians (*approbirten Aerzten*) who have registered their names and obtained their membership-cards. Other savans, who are interested in the work of the congress, may be admitted as extraordinary members."

The delegates did not see fit to change this article so as to include dental surgeons, but decided that the article should be so interpreted as to admit dentists to membership. Since the meeting at Heidelberg the question has been raised whether dentists resident in Germany, but not possessing the German dental approbation (degree), could be admitted to membership. Regarding this point the chairman of the committee on organization decided that only those who possess the recognized degree of that country of which they are citizens may be admitted to membership.

A German citizen holding only an American or Swiss degree is, therefore, not entitled to membership, no more is an American or English citizen not possessing the degree of his own country; on the other hand, foreign citizens practising in Germany are admitted without the German degree, provided they have the degree of their own country.

Members pay a fee of twenty marks ($5.00), and receive a copy of the transactions.

Art. III. The object of the congress is exclusively scientific.

Art. X. All lectures and communications in the general sittings, or in those of the sections, must be handed in in writing to the secretary before the close of the sitting. The Editorial Committee decides whether, or in what part, such communications shall be included in the published transactions.

Art. XI. The official languages of all sittings are German, English, and French. Very short remarks may be made in other languages, provided some member is prepared to translate them into one of the official languages.

Art. XII. Sections are, as a rule, to be limited to twenty minutes; discussional remarks to ten minutes.

Art. XIV. Students of medicine and other persons, gentlemen and ladies who are not physicians, but are interested in the proceedings of any particular session, may be invited by the president of that session, or, on application, receive permission to attend as auditors.

There are to be no vice-presidents associated with the congress, but each section is empowered to elect a limited number of honorary presidents and a secretary for each of the official languages.

The committee of the Dental Section, No. 14, is composed as follows: Busch, Berlin, chairman; Calais, Hamburg; Hesse, Leipsic; Fricke, Kiel; Holländer, Halle; Miller, Berlin; Partsch, Breslau; Sauer, Berlin; Weil, Munich.

At a meeting of this committee, held on the 16th of October, 1889, it was decided that the hours from 9 to 12 A.M. should be devoted to practical demonstrations in the rooms of the dental institute, the demonstrations to consist of operations in filling, extraction, and in mechanical dentistry,—in short, operations in all branches of operative and mechanical dentistry.

Demonstrations in extraction and in artificial work are to be under the direction of Professor Busch, those in filling under that of Professor Miller. The theoretical exercises, etc., are to be held from 2 to 5 P.M. They will consist of the usual essays or lectures and the accompanying discussions; besides these, three subjects for general discussion are to be chosen, one to be introduced in the German language (on bromide of ethyl, by Professor Holländer), one in the English, and one in the French languages.

Those desiring to deliver lectures or read essays on particular subjects are requested to send in, along with their announcement, a very short *résumé* of the contents of the same.

Correspondence in German language to be directed to Professor Busch, chairman, No. 40 Dorotheen Street, Berlin; in French language to Dr. Calais, No. 17 Hohenbleichen Street, Hamburg; in English to Professor Miller, No. 32 Voss Street, Berlin.

In America, Drs. Barrett and Taft; in Great Britain, Mr. J. H. Mummery, M.R.C.S., etc., and Mr. W. Bowman Macleod, F.R.S.E., etc., have, on invitation by the committee, expressed their willingness to act in the capacity of honorary presidents.

A fuller report of the steps taken in the organization of the congress, up to the end of October, is given by Professor Busch in the *Verhandlungen der deutschen odontologischen Gesellschaft*, Heft 2.

W. D. MILLER.

